# Do syntopic host species harbour similar symbiotic communities? The case of *Chaetopterus* spp. (Annelida: Chaetopteridae)

**DOI:** 10.7717/peerj.2930

**Published:** 2017-02-02

**Authors:** Temir A. Britayev, Elena Mekhova, Yury Deart, Daniel Martin

**Affiliations:** 1 Severtzov Institute of Ecology and Evolution, Russian Academy of Sciences, Moscow, Russian Federation; 2 Department of Marine Ecology, Centre d’Estudis Avançats de Blanes (CEAB–CSIC), Blanes, Catalunya, Spain

**Keywords:** Symbiotic community structure, Polychaeta, Carapidae, Porcellanidae, Pinnotheridae, Tergipedidae, Competition, South China Sea, Vietnam

## Abstract

To assess whether closely related host species harbour similar symbiotic communities, we studied two polychaetes, *Chaetopterus* sp. (*n* = 11) and *Chaetopterus* cf. *appendiculatus* (*n* = 83) living in soft sediments of Nhatrang Bay (South China Sea, Vietnam). The former harboured the porcellanid crabs *Polyonyx* cf. *heok* and *Polyonyx* sp., the pinnotherid crab *Tetrias* sp. and the tergipedid nudibranch *Phestilla* sp. The latter harboured the polynoid polychaete *Ophthalmonoe pettiboneae*, the carapid fish *Onuxodon fowleri* and the porcellanid crab *Eulenaios cometes*, all of which, except *O. fowleri*, seemed to be specialized symbionts. The species richness and mean intensity of the symbionts were higher in *Chaetopterus* sp. than in *C.* cf. *appendiculatus* (1.8 and 1.02 species and 3.0 and 1.05 individuals per host respectively). We suggest that the lower density of *Chaetopterus* sp. may explain the higher number of associated symbionts observed, as well as the 100% prevalence (69.5% in *C.* cf. *appenciculatus*). Most *Chaetopterus* sp. harboured two symbiotic species, which was extremely rare in *C.* cf. *appendiculatus*, suggesting lower interspecific interactions in the former. The crab and nudibranch symbionts of *Chaetopterus* sp. often shared a host and lived in pairs, thus partitioning resources. This led to the species coexisting in the tubes of *Chaetopterus* sp., establishing a tightly packed community, indicating high species richness and mean intensity, together with a low species dominance. In contrast, the aggressive, strictly territorial species associated with *C.* cf. *appendiculatus* established a symbiotic community strongly dominated by single species and, thus, low species richness and mean intensity. Therefore, we suggest that interspecific interactions are determining species richness, intensity and dominance, while intraspecific interactions are influencing only intensity and abundance. It is possible that species composition may have influenced the differences in community structure observed. We hypothesize that both host species could originally be allopatric. The evolutionary specialization of the symbiotic communities would occur in separated geographical areas, while the posterior disappearance of the existing geographical barriers would lead to the overlapped distribution.

## Introduction

During the last few decades, significant efforts have been undertaken to study the species composition and structure of marine symbiotic communities associated with different hosts taxa such as scleractinian corals (Hoeksema, Van der Meij & Fransen, 2012; Stella, Jones & Pratchett, 2010), echiurans (Anker et al., 2005), hermit crabs (Williams & McDermott, 2004) and echinoderms (Barel & Kramers, 1977). Despite this being an interesting aspect of marine ecosystems’ functioning and the need to fill in existing gaps in related knowledge, the current focus of scientific interests have shifted to ecological and evolutionary aspects of the establishment of symbiotic communities (Baeza, 2015; Duffy, 2002; Thiel & Baeza, 2001). Accordingly, host characteristics (morphological, ecological and physiological) have been considered as some of the most important parameters driving these processes (e.g., Abele & Patton, 1976; Deheyn, Lyskin & Eeckahaut, 2006; Goto & Kato, 2011).

The coexistence of potential hosts that are taxonomically closely related (thus sharing similar morphological and physiological characteristics) may facilitate host switching, leading to the infestation of different host species by the same species of symbiont, as reported for example in freshwater fish (Poulin, 1998). Accordingly, we may expect the composition of symbiotic communities established on closely related hosts to be similar. Hence, sympatric coral species belonging to the same family harbour symbiotic communities more similar than those belonging to different families (Stella, Jones & Pratchett, 2010), while the symbiotic communities associated with two starfish hosts from the same family living in the same area have nearly identical species composition (Antokhina, Savinkin & Britayev, 2012). There seems to be a correlation between increasing taxonomic proximity between hosts and a higher similarity in species composition of the respective symbiotic communities. In other words, we could expect that closely related (i.e., belonging to the same genus) host species sharing the same habitat would harbour very similar (or even identical) symbiotic communities. Therefore, the current study investigated the symbiotic communities associated with two species of *Chaetopterus* in Nhatrang Bay (Vietnam), to assess whether this hypothesis may apply to this particular situation.

These two species of *Chaetopterus* appeared to be excellent subjects for the intended comparison due to their highly similar morphology. In fact, the genus has long been regarded as monospecific and, to date, the morphological identification of species is still considered as rather complex (Britayev & Martin, 2016; Nishi, Hickman Jr & Bailey-Brock, 2009; Petersen, 1984a; Petersen, 1984b). Moreover, these two species share the same habitat and, thus the influence of environmental parameters can be excluded as influential factors on the associated symbiotic communities.

The genus *Chaetopterus* (Annelida: Chaetopteridae) includes relatively large animals (up to 20–25 cm in length) living in roughly U-shaped tubes embedded into soft sediments or attached to hard surfaces in shallow waters of temperate and tropical seas (Britayev & Martin, 2016). Morphologically, they are highly adapted for feeding on plankton using complex mucus-net based mechanisms (Enders, 1909). They are also well known as hosts harbouring numerous symbiotic associates (often including complex communities) inside their parchment-like tubes. These tubes provide well-protected shelter with continuous water flow bringing oxygen and food items to the symbionts (Britayev & Martin, 2016). To date, approximately 28 species of symbionts have been reported living inside tubes of *Chaetopterus* (Petersen & Britayev, 1997). However, information on the composition of associated communities is lacking, and is currently only available for two species, *C. pergamentaceus* Cuvier, 1830 and *C.* cf. *cautus* Marenzeller, 1879, which are each host to 3–5 species of crabs and polychaetes (Britayev, 1993; Gray, 1961).

A species of *Chaetopterus* (not confirmed but probably *Chaetopterus* cf. *appendiculatus* Grube, 1874) inhabiting Vietnamese soft seabed sediments was previously reported as harbouring three species of symbionts within its tubes: the polychaete *Ophthalmonoe pettiboneae* Petersen & Britayev, 1997, an unidentified carapid fish and a porcellanid crab (Britayev & Martin, 2005). The presence of a second, probably undescribed, species of *Chaetopterus* sharing the same habitat and having its own associated symbiotic community allowed us to investigate the hypothesis that postulates the similarity in composition of symbiotic communities associated with morphologically similar hosts.

More specifically, in this paper we analyse: (1) The morphological and ecological characteristics of the two Vietnamese host species of *Chaetopterus*; (2) The composition, species richness and abundance of the symbiotic communities associated with the two host species; and (3) The host specificity of all symbiotic species.

## Material and Methods

**Figure 1:**
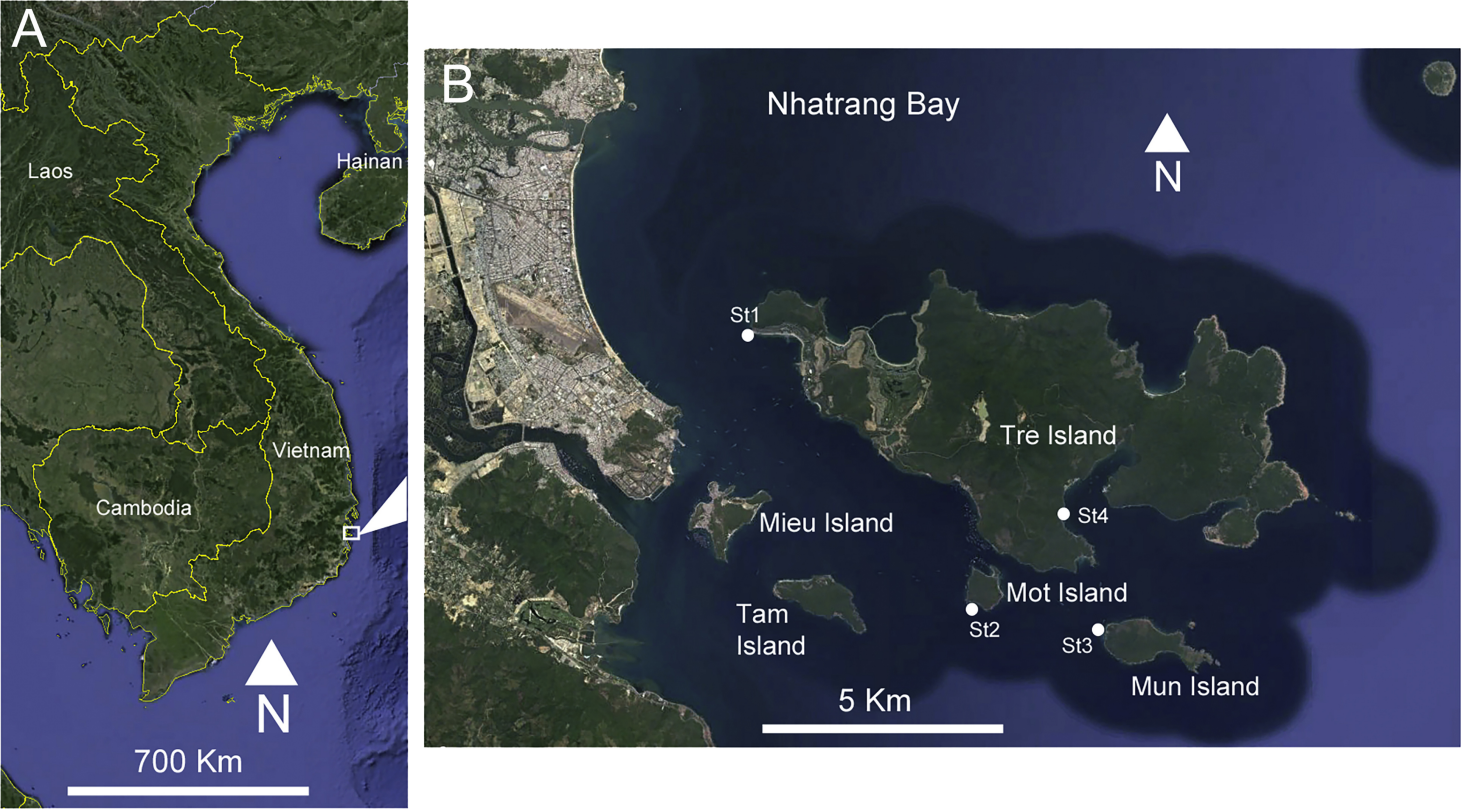
(A) Location of the study area on the Vietnamese coast of the South China Sea. (B) Sampling sites, Nhatrang Bay. Map data: SIO, NOAA, US Navy, NGA, GEBCO. Image (C) 2016 Digital Globe and Google Earth.

Sampling was conducted between March and April 2016 in four localities of Nhatrang Bay (Vietnam, South China Sea): the western coast of Mun Island, the southern coast of Mot Island, the western coast of Tre Island and Dam Bay (Fig. 1, Table 1). The Russian-Vietnamese Tropical Center issued a letter supporting the collection of samples and animals used in this paper.

**Table 1:** Depth (m) and geographical coordinates of the studied locations, as well as density (chaetopterid individuals per 100 m^2^/per diving hour), ratio of individuals (*Chaetopterus* sp. vs. *Chaetopterus* cf. *appendiculatus*) and infestation prevalence (%) of the respective host populations

Locality	Station	Latitude	Longitude	Depth	Density	Ratio	Prevalence
Mun Island	1	12°10′10″N	109°17′46″E	13–16	1.0/3.8	2/20	65.2
Dam Bay	2	12°11′43″N	109°17′26″E	6–8	0.6/3.0	2/23	81.8
Mot Island	3	12°10′26″N	109°16′23″E	16–20	nd/4.7	7/20	90.9
Point Nam Tre Island	4	12°13′42″N	109°13′47″E	10–12	nd/7.1	1/19	60.0

**Notes.**

nd no data

 The chaetopterid hosts were collected by SCUBA diving at depths from 6 to 20 m. As their tubes were embedded within the sediment to 15–20 cm depth, extraction was achieved by washing out the sediments by hand. The tubes were then gently removed, immediately placed into individual zip-lock plastic bags to avoid losing symbionts and transferred to seawater tanks, where they were kept until reaching the laboratory facilities.

The density of the studied population of *Chaetopterus* was estimated along five 50 m long and 2 m wide transects at Mun Island and Dam Bay. At each site, the transects followed the depth profile and were placed parallel, each one immediately adjacent to the previous one. Two divers were responsible for counting the number of chaetopterid tube siphons, each one on one side (1 m) of the transect. A second density estimate was based on the number of chaetopterids sampled per hour at each sampling site (except when diving surveys were used for transect estimates).

In the laboratory, tube length was measured to the nearest 5.0 mm (Table S1). Then, tubes were gently opened by hand and carefully checked for presence of symbionts. The species and number of symbionts were recorded (Table S1). Water and sediment from the bag were sieved through a 1 mm mesh and the retained sediments were carefully inspected by eye. The body in *Chaetopterus* is divided into three differentiated regions: the nine-to-ten anterior-most segments, the five mid-body segments, and an undefined (but usually very numerous) number of posterior segments, which form the regions A, B, and C, respectively (Britayev & Martin, 2016). Hosts were extracted and measured either as length and width for region A (*n* = 8) or as displaced water volume in a graduate vessel to the nearest 1 ml (all remaining specimens) (Table S1). As body volume showed a positive linear relationship with tube length (Tube length = 44.084 + 0.503*Body volume, *F* = 26.457, *P* < 0.0001; Table S2), this easy-to-obtain measurement was used to study community structure.

All chaetopterid tubes, hosts and symbionts were photographed with Canon digital cameras (G16 and EOS 6D). Selected hosts and all symbionts were fixed either in 70% or 99% ethanol, or in a 4% formaldehyde/seawater solution for further studies. Small fragments of the ventral uncinal tori of both host species have been dissected. To illustrate the shape of the uncini, these fragments were squashed between slides, mounted in glycerine and photographed with the help of a ProgRes C10 Plus digital camera (Jenoptics, Jena) attached to a Zeiss Axioplan compound microscope.

All symbionts were measured to the nearest 0.1 mm, using a calibrated ocular micrometer under an Olympus SZX9 stereomicroscope as body length from tip of prostomium to the end of pygidium for polychaetes, as body length from tip of head to the end of caudal peduncle for fish, and as carapace width for crabs. Crabs were sexed according to the abdominal shape.

DNA was extracted using Spin Columns Thermo Scientific GeneJET 50 kit, following the manufacturer’s standard protocol. 10 ng of genomic DNA was used as a template for polymerase chain reaction (PCR) with special mitochondrial Cox1 primers: TGTAAAACGACGGCCAGTGAYTATWTTCAACAAATCATAAAGATATTGG and CAGGAAACAGCTATGACTAMACTTCWGGGTGACCAAARAATCA (Carr et al., 2011). PCR were set up in total volume of 20 µl. The PCR cycling profiles were as follows: initial denaturation (95 °C, 5 min); followed by 35 cycles of denaturation (95 °C, 15 s), annealing (45 °C, 15 s) and extension (72 °C, 60 s). The resulting PCR products were purified by direct purification from the PCR mixture and prepared for sequencing. Overlapping sequence fragments were merged into consensus sequences using MEGA7 (Kumar, Stecher & Tamura, 2016), the protein coding COI being simple to align. The obtained COI sequences and voucher paragenophores (Pleijel et al., 2008) for the two species of *Chaetopterus* have been deposited in GenBank and in the collections of the Severtsov Institute of Ecology and Evolution RAS, respectively. Seven host specimens were used in genetic analyses to ensure species delineation (Table 2). The genetic differentiation within and between species was assessed by pairwise genetic distances between COI sequences using the Maximum Likelihood Model, which allowed us to show the percentage of replicate trees in which the associated taxa clustered together in the bootstrap test (1,000 replicates) next to each branch (Felsenstein, 1985). The phylogenetic tree was drawn to scale, with branch lengths in the same units as those of the evolutionary distances used to infer it, as computed using the Maximum Composite Likelihood method (Tamura, Nei & Kumar, 2004) and are in the units of the number of base substitutions per site. The phylogenetic tree was built using the COI sequences of *Chaetopterus* and *Mesochaetopterus* available from NCBI GenBank, using *Spiochaetopterus costarum* (Claparéde, 1869) as the outgroup, by means of the Neighbour-Joining method (Saitou & Nei, 1987) in MEGA7 (Kumar, Stecher & Tamura, 2016).

**Table 2:** Specimens list for the two Vietnamese host species used in the molecular analyses, detailing the GenBank accession numbers and the collection references for the voucher paragenophores.

*Chaetoperus*	Specimen number	Accession number	Voucher
cf. *appendiculatus*	14	KY124465	sevin Pl/Vn 2016Ch0001
cf. *appendiculatus*	76	KY124466	sevin Pl/Vn 2016Ch0002
cf. *appendiculatus*	77	KY124467	sevin Pl/Vn 2016Ch0003
cf. *appendiculatus*	80	KY124468	sevin Pl/Vn 2016Ch0004
sp.	16	KY124469	sevin Pl/Vn 2016Ch0005
sp.	82	KY124470	sevin Pl/Vn 2016Ch0006
sp.	93	KY124471	sevin Pl/Vn 2016Ch0007

For the purposes of our study, the following terms are defined: Prevalence, as the ratio between number of infested and total number of hosts; Intensity, as the number of symbionts present in each infested host; Mean intensity, as the mean number of individuals of a particular symbiotic species per infested host in a sample; Abundance, as mean number of symbionts per examined host, infested and non-infested; and Species richness, as mean number of symbiotic species per infested host.

The porcellanid crabs were identified by Prof. Bernd Werding, from the Institut für Allgemenie und Spezielle Zoologie of the Justus-Liebig Universität (Giessen, Germany). The pinnotherid crab was identified by Prof. Peter Ng from the Department of Zoology of the National University of Singapore (Republic of Singapore). The carapid fish was identified by Dr. Eric Parmentier from the Laboratoire de Morphologie Fonctionnelle et Evolutive of the Institut de Chimie of the Université de Liege (Belgium). The tergipedid nudibranch was identified by Dr. Irina Ekimova, from the Department of Invertebrate Zoology of the Lomonosov Moscow State University (Russian Federation).

The relationship between host body volume and tube length were assessed by linear regression. The species richness and mean intensity, as well as the average length of infested and non-infested tubes of *Chaetopterus*, were compared by Student’s *t*-test. Statistical analyses were performed using Statistica 6.0 and PAST 2.17 software.

## Results

### Hosts characteristics

**Figure 2:**
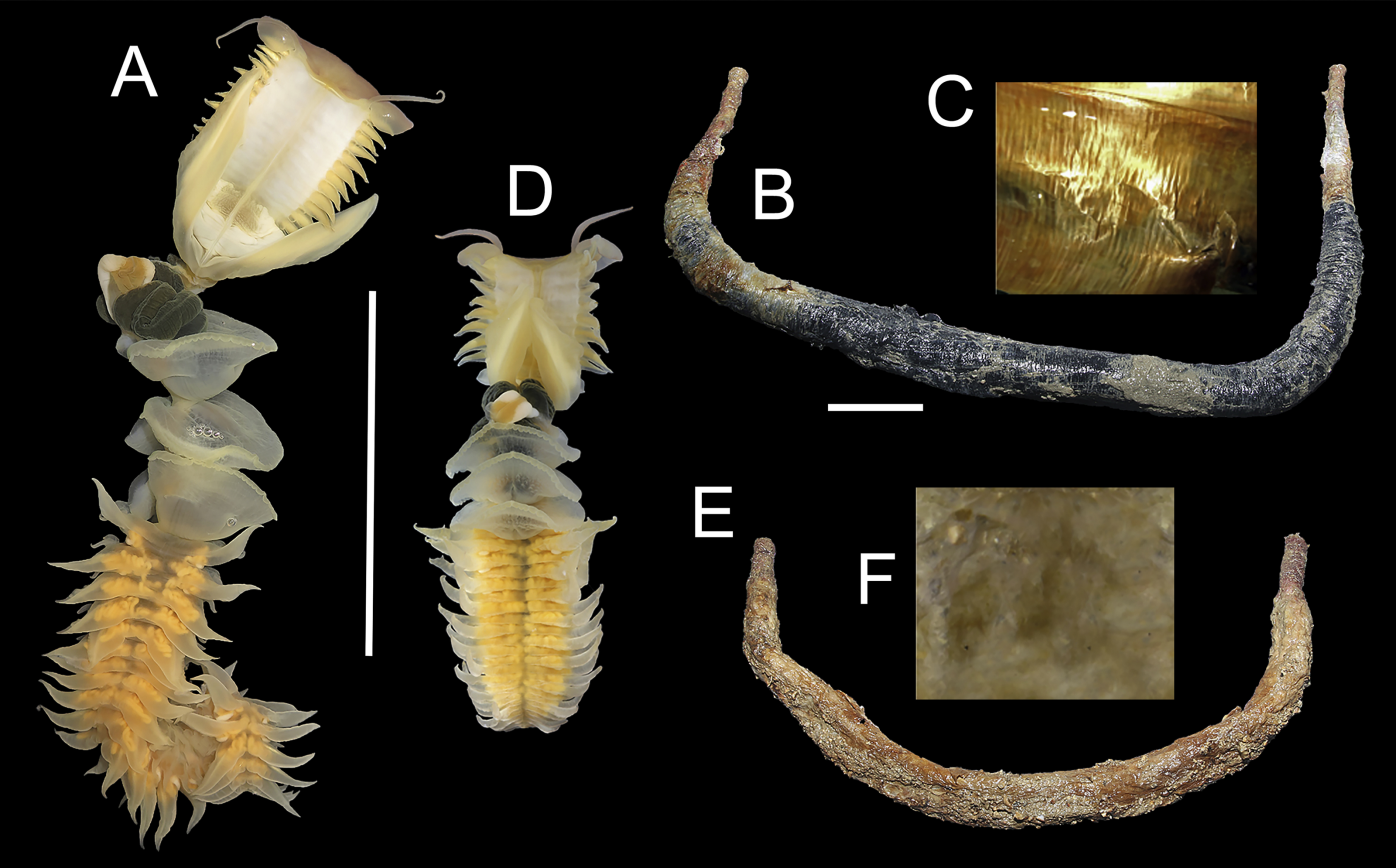
*Chaetopterus* cf. *appendiculatus*: (A) whole worm; (B) tube; (C) detail of inner tube surface. *Chaetopterus* sp.: (D) whole worm; (E) tube; (F) detail of inner tube surface. Scale bars are 5 cm.

**Figure 3:**
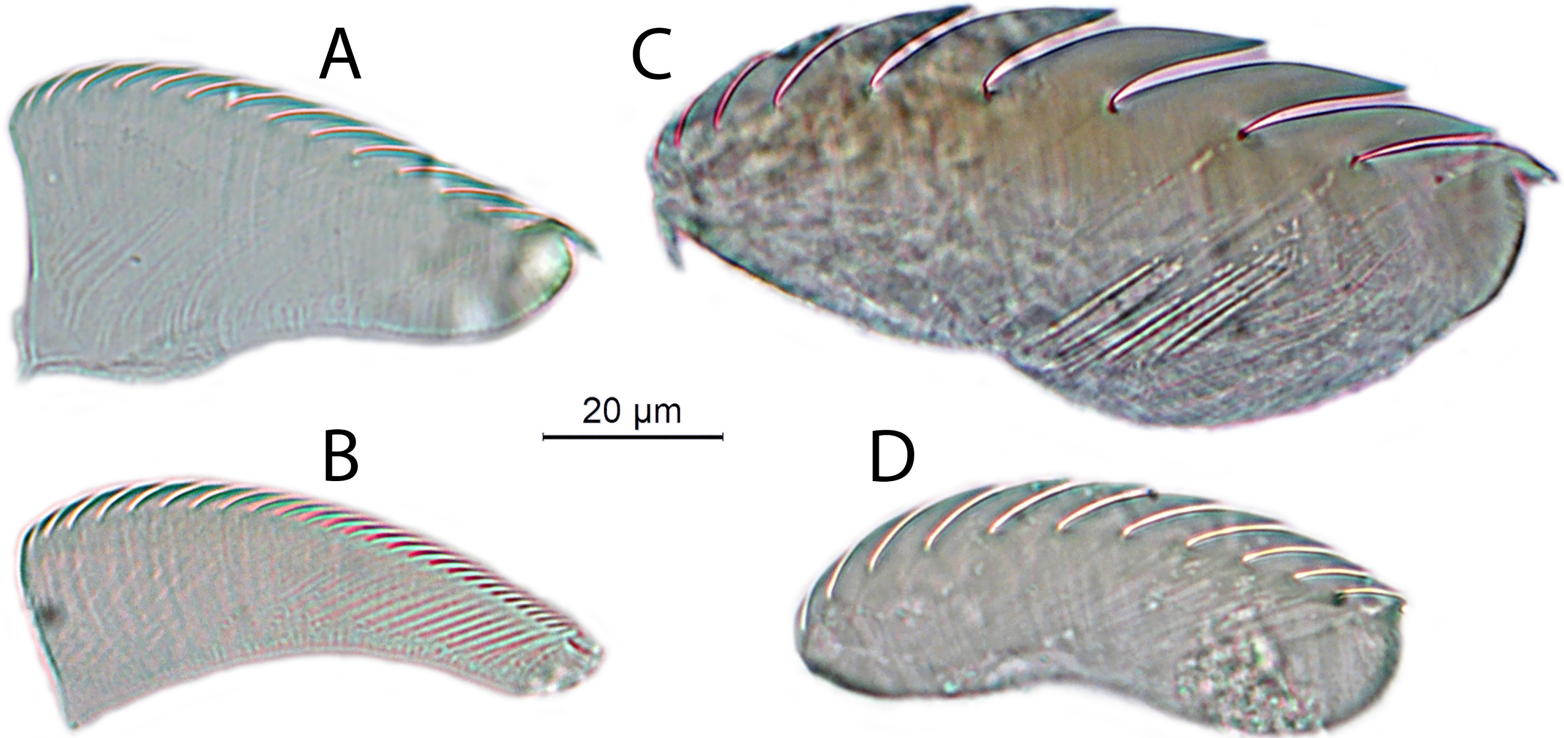
Uncini from ventral neuropodial tori of region C. *Chaetopterus* cf. *appendiculatus*: (A) upper tori; (B) lower tori. *Chaetopterus* sp.: (C) upper tori; (D) lower tori.

The two Vietnamese host species of *Chaetopterus* are morphologically similar. However, one of them is significantly bigger than the other, both in terms of tube length (1.4:1, on average) and body volume (2.7:1, on average) (*t*-test *p* < 0.0001, Table 3). They also differ in the number of chaetigers of region A (9 and 9–11, respectively) (Figs. 2A and 2D) and in the denticles of the neuropodial uncini of region C (25–35 and 9, respectively) (Figs. 3A–3D), as well as in tube structure. Tubes of the bigger species are covered by silt, have a parchment-like appearance and the inner lining is iridescent, silver or golden in colour, showing distinct transverse annulations (Fig. 2C). In the smaller species, tubes are covered by sand and small coral and shell fragments, have a paper-like appearance with a semi-transparent inner lining, whitish or brownish in colour and lacking distinct annulations (Fig. 2F).

**Table 3:** Number of individuals, mean tube length (min–max), cm and mean body volume (min–max) of *Chaetopterus* cf. *appendiculatus* and *Chaetopterus* sp.

Species	Number	Tube length (cm)	Body volume (cm^2^)
*Chaetopterus* cf. *appendiculatus*	83	64.6 (41–88)	41.9 (23–72)
*Chaetopterus* sp.	11	44.8 (23–58)	15.8(2–32)

We tentatively identified the bigger host species as *Chaetopterus* cf. *appendiculatus* because, according to the original description, this species has a sand-coloured inner tube surface, showing dense transverse annulations. Additionally, it is the only described species of *Chaetopterus* possessing neuropodial uncini from region C with more than 20 small denticles. *Chaetopterus appendiculatus* was already reported as host of *O. pettiboneae* from the Banda Sea (Indonesia) by Petersen & Britayev (1997). Petersen (1997) proposed the redescription of *C. appendiculatus* as a valid species, based on the type material from Ceylon. The fact that formal redescription has never been published does not prevent us from considering the species as valid, whose formal redescription is far beyond the scope of this paper. However, the long geographical distance between Ceylon/Indonesia and Vietnam prevents us in fully assigning the Vietnamese specimens to *C. appendiculatus*, and we refer to the species as *C*. cf. *appendiculatus* in this paper. The smaller host is likely  undescribed.

**Figure 4:**
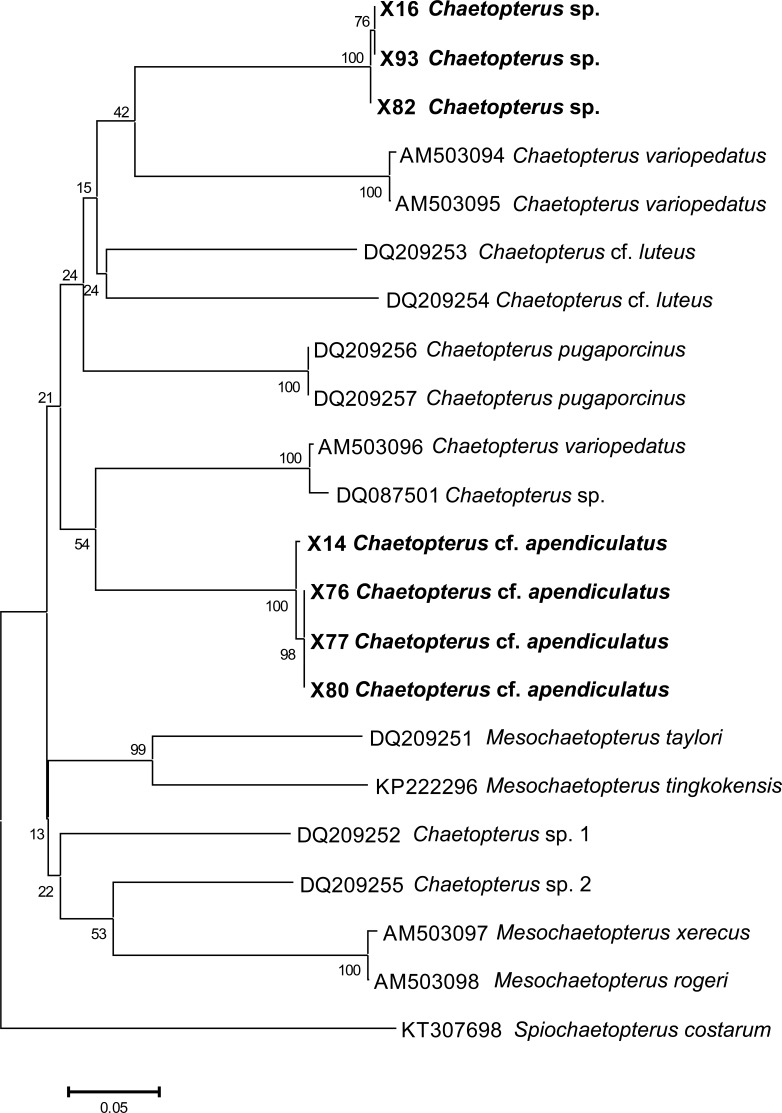
Preliminary phylogenetic tree for species of *Chaetopterus* and *Mesochaetopterus* based on the COI sequences obtained from NCBI GenBank and our data. The sequences for the two Vietnamese species are listed in Table 2.

The phylogenetic analysis including the COI sequences of the Vietnamese hosts (Fig.  4) showed low bootstrap values that did not allow us to fully resolve the phylogeny of *Chaetopterus*. However, it clearly revealed that the two Vietnamese hosts are different species included within two separate monophyletic clades (with 100% bootstrap support), thus confirming our morphological inference. Although with low support, the closest clades to those of the two Vietnamese *Chaetopterus* belong to *C. variopedatus* (Renier, 1804). However, the specimens joining the *Chaetopterus* sp. clade (42% bootstrap support) originate from the Mediterranean, while those joining the *C*. cf. * appendiculatus* clade (54% bootstrap support) originate from the Atlantic. As indicated by Martin et al. (2008), our results support the inference that the two populations of *C. variopedatus* belong to different species, with the Mediterranean species described and the Atlantic species still undescribed. The results also confirm that *C. variopedatus* sensu Hartman (1959) is not a single cosmopolitan species, but a complex including more than 20 different species (Bhaud, 1998; Osborn et al., 2007; Petersen, 1984a; Petersen, 1984b; Petersen, 1997). As is the case for *C. appendiculatus*, some of these species have not yet been formally redescribed. However, as many as nine species have recently been described, and five have been redescribed in the recent literature (Nishi, 2001; Nishi, Arai & Sasanuma , 2000; Nishi, Hickman Jr & Bailey-Brock, 2009; Osborn et al., 2007; Sun & Qiu, 2014).

The two Vietnamese *Chaetopterus* host species were found at the same localities, with their tubes deeply embedded in silty sand sediments. *Chaetopterus* cf. *appendiculatus* outnumbered *Chaetopterus* sp. in all samples, yet their proportion varied depending on the locality, with *Chaetopterus* sp. being relatively more abundant at Mot Islands (St. 2, 25.9%) and substantially less abundant at the other stations (5.0–9.1%) (Table 1).

The density of *Chaetopterus* ranged from 0.6 to 1.0 individuals per 100 m^2^ in the transects, while the number of collected worms per diving hour was lower at St. 4 in Dam Bay and higher at St. 1 in Point Nam, Tre Island (Table 1).

### Taxonomic composition of the symbiotic communities

**Figure 5:**
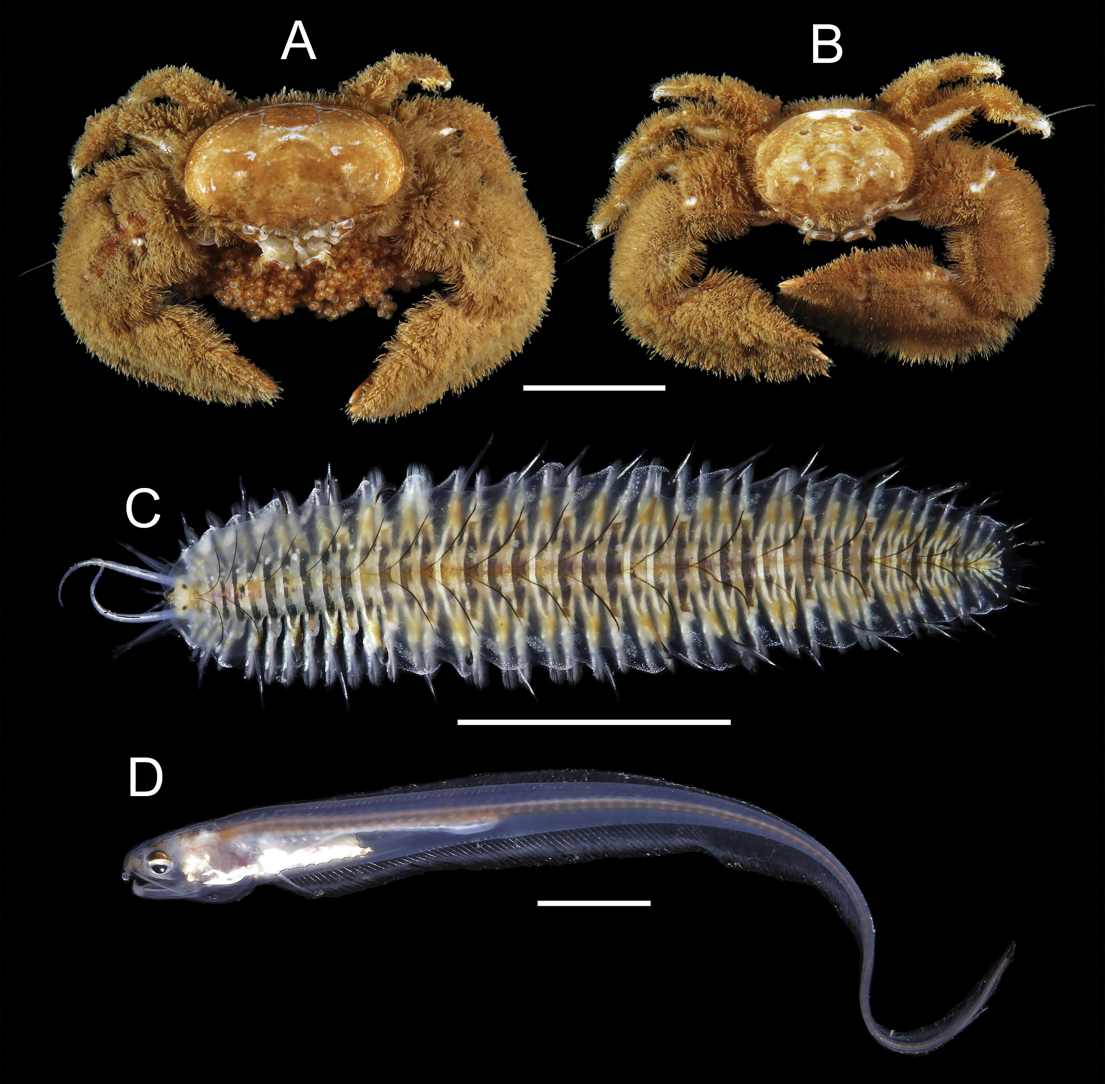
Symbiotic species associated with *Chaetopterus* cf. *appendiculatus*: (A, B) *Eulenaios cometes* (female and male, respectively); (C) *Ophthalmonoe pettibonneae*; (D) *Onuxodon fowleri*. Scale bars are 1  cm.

**Figure 6:**
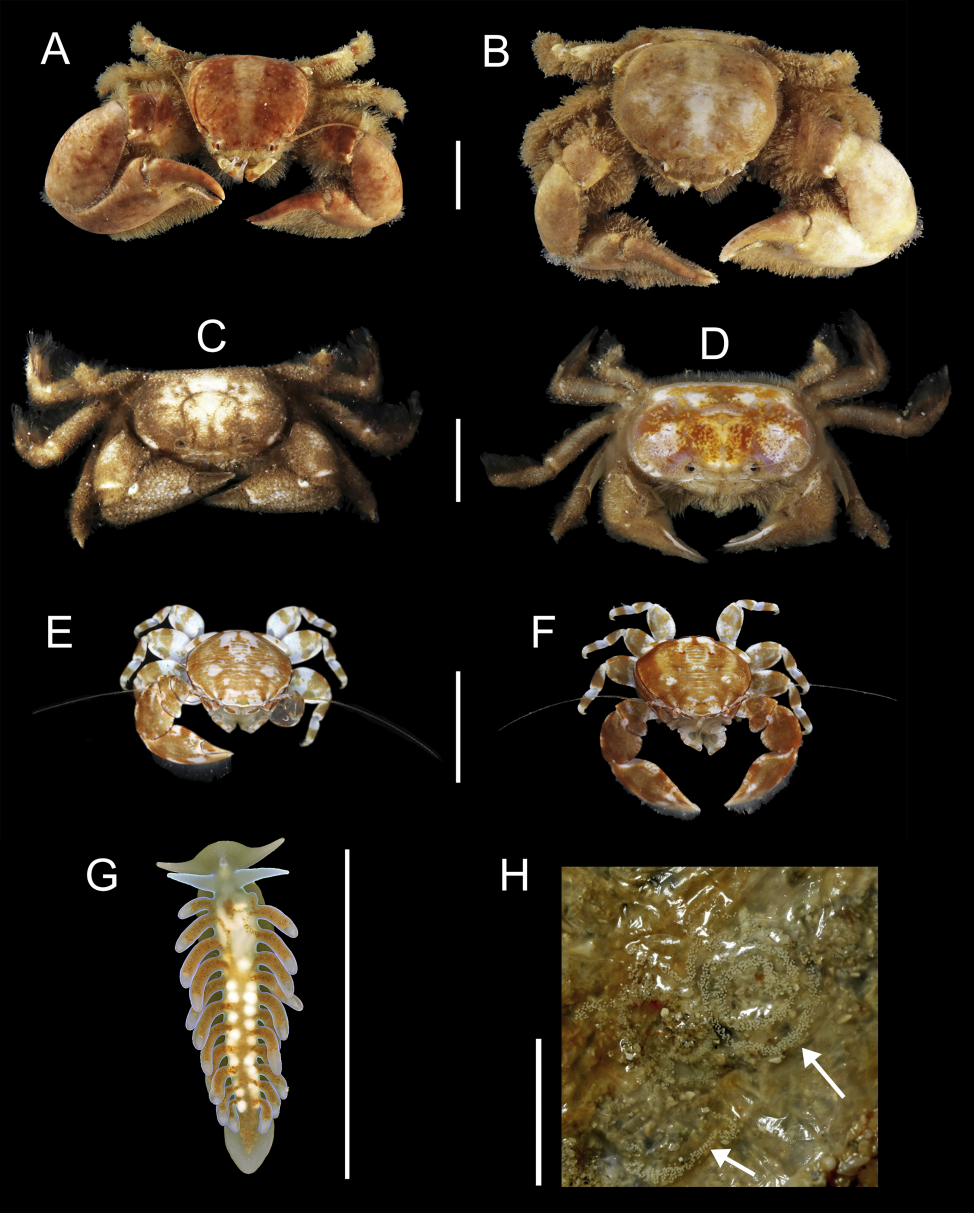
Symbiotic species associated with *Chaetopterus* sp.: (A, B) *Polyonyx* cf. *heok* (male and female, respectively); (C, D) *Tetrias* sp. (male and female, respectively); (E, F) *Polyonyx* sp., (male and female, respectively); (G, F) *Phestilla* sp. (whole body and egg-mass, respectively); egg-mass indicated by arrows. Scale bars are 0.5 cm.

91 individuals of seven species of animals occurred in association with the two host species of *Chaetopterus*. Among them, the polynoid polychaete *Ophthalmonoe pettiboneae* (Fig. 5C), the tergipedid nudibranch *Phestilla* sp. (Fig. 6G), the carapid fish *Onuxodon fowleri* (Smith, 1964) (Fig. 5D), and four species of decapods, three porcellanids, *Eulenaios cometes* (Walker, 1887) (Figs. 5A and 5B), *Polyonyx* cf. *heok* (Osawa & Ng, 2016) (Figs. 6A and 6B) and *Polyonyx* sp. (Figs. 6E and 6F), and the pinnotherid *Tetrias* sp. (Figs. 6C and 6D) (Table 4).

**Table 4:** Prevalence (%) and mean intensity (mean number of individuals per infested host) of the symbiotic species associated with the two host *Chaetopterus*.

Symbiont species	*Chaetopterus* sp.	*Chaetopterus* cf. *appendiculatus*
*Ophthalmonoe pettiboneae* (P)	–	64.1 (1.0)
*Phestilla* sp. (G)	22.2 (2.0)	–
*Eulenaios cometes* (D)	–	1.3 (2.0)
*Polyonyx* cf. *heok* (D)	88.9 (1.7)	–
*Polyonyx* sp. (D)	66.7 (1.3)	–
*Tetrias* sp. (D)	11.1 (2.0)	–
*Onuxodon fowleri* (A)	–	6.4 (1.2)

**Notes.**

P Polychaeta D Decapoda G Gastropoda A Actinopteri

**Figure 7:**
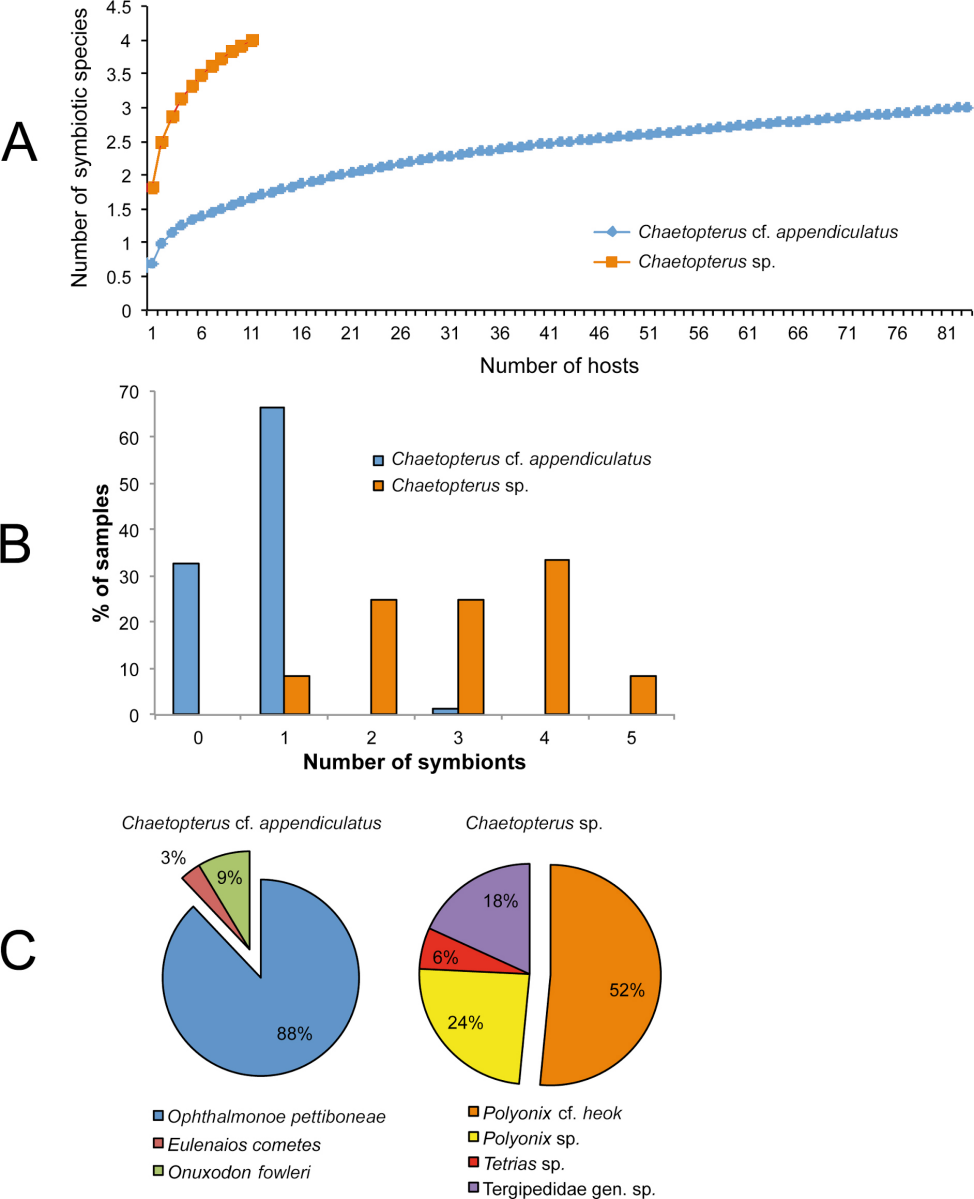
Characterization of the symbiotic communities associated with the two host species of *Chaetopterus*: (A) rarefaction curve; (B) distribution of symbionts per host; (C) relative abundance of the symbiotic species.

Four and three species were found inside the tubes of *Chaetopterus* sp. and *C*. cf. *appendiculatus*, respectively. Surprisingly, the symbiotic communities associated with the two hosts did not have any species in common, with the only similarity at a higher taxonomic level being the presence of porcellanid crabs (Table 4). Despite the lower sample size of *Chaetopterus* sp., the diversity of its associated community was higher than that of *C*. cf. *appendiculatus.* Accordingly, it may be expected that the number of species associated with *Chaetopterus* sp. would increase with an increasing number of analysed host individuals. Conversely, the diversity of the community associated with *C*. cf. *appendiculatus* showed an almost saturated species accumulation curve (Fig. 7A).

*Ophthlmonoe pettiboneae* is the single symbiotic species previously known from Vietnamese waters and from the same host species. The other six are here reported for the first time from the Vietnamese coasts. Moreover, *O. fowleri* is herein reported as a symbiont of chaetopterids for the first time, as well as *Tetrias* sp., *Polyonyx* sp., *P*. cf. *heok* and *Phestilla* sp., which are new to science and will be described at later dates in specialized papers. The tergipedid nudibranch is also, to the best of our knowledge, the first known nudibranch living in symbiosis with a polychaete host. It shows a posterior end functioning as a sucker (Fig. 6F) allowing it to attach to the smooth inner surface of the host tube, while the rest of the body can move freely (https://www.researchgate.net/publication/310159685_Phestilla_sp). Its flattened body, together with the lack of cnidosacs and the uniserial radula with long lateral denticles on the rachidian tooth clearly place it within the genus *Phestilla*. However, it differs from all known species of this genus by having a small central denticle of the radula, a wider foot and cerata arranged one per row only (Y Deart & I Ekimova, pers. comm., 2016). Moreover, its appearance and colouring (Fig. 6F) mimics, to some extent, that of the very posterior end of the chaetopterid host.

### Structure of the symbiotic communities

A total of 61 (73.5%) out of 83 individuals of *C*. *appediculatus* and all 11 (100%) *Chaetopterus* sp. were infested. Among the species associated with *C*. *appediculatus*, *O. pettiboneae* showed a higher prevalence than the two other symbionts (Table 4). Among the associates with *Chaetopterus* sp., the prevalence ranged from 11% to almost 90%, with the maximum corresponding to *P*. cf. *heok* (Table 4).

The number of species inhabiting the same tube varied from zero to two in *C*. cf. *appendiculatus*, and from one to two in *Chaetopterus* sp. However, the species richness was significantly higher in the latter (*p* > 0.001, Figs. 7A and 7B) due to the common coexistence of two symbiont species in the same host tube. In fact, the small-sized *Polyonyx* sp. and *Phestilla* sp. (Figs. 6E and 6G) were found in all observed cases living with other crab species, mostly with the large sized *P*. cf. *heok* (Figs. 6A and 6B). Only in one case, *Polyonyx* sp. shared the host tube with *Tetrias* sp. (Table 5). In contrast, most tubes of *C*. *appediculatus* were occupied by a single symbiotic species, either *O. pettiboneae* or *O. fowleri*. Only in one case two species of symbionts (*O. pettiboneae* and *E. cometes*) were present sharing the same host tube (Table 5).

**Table 5:** Distribution among hosts (as number of host tubes found without and with one, two and three individuals) for the five species associated with *Chaetopterus* spp.

Symbiont	*Chaetopterus*	0	1	2	3	4
*Ophthalmonoe pettiboneae*	Ca	21	56	0	0	0
*Onuxodon fowleri*	Ca	78	5	1	0	0
*Eulenaios cometes*	Ca	8	0	1	0	0
*Polyonyx* cf. * heok*	Cs	1	3	7	0	0
*Polyonyx* sp.	Cs	3	4	2	0	0
*Tetrias* sp.	Cs	10	0	1	0	0
*Phestilla* sp.	Cs	8	1	1	1	0

**Notes.**

Cs *Chaetopterus* sp. Ca *Chaetopterus* cf. *appendiculatus*

The number of symbiont individuals infesting one host varied from zero to three in *C*. cf. *appediculatus*, and from one to five in *Chaetopterus* sp., while the mean intensity was nearly three times lower in the former than in the later (*p* > 0.001, Table 6). Accordingly, most *C*. cf. *appediculatus* were infested by one symbiotic individual, while multiple infestation (two, three, or even four symbionts) was common in *Chaetopterus* sp. (Fig. 7B).

**Table 6:** Symbiotic community indexes: species richness (mean number of species per one infested host), infestation prevalence (%), and mean intensity (mean number of individuals per infested host).

	Richness	Prevalence	Intensity
*Chaetopterus* sp.	1.8	100	3.0
*Chaetopterus* cf. *appendiculatus*	1.02	73.5	1.05

The distribution pattern of the symbionts among their hosts was primarily regular, as all. *O. pettiboneae* and most *O. fowleri* lived solitary inside their host tubes. In turn, *Polyonyx* cf. *heok, Polyonyx* sp., *E. cometes, Tetrias* sp. were found in host tubes usually as male/female pair. The number of nudibranchs varied from one to three per hosts (Table 6) and, in one case, a couple was observed near to a recently spawned egg-mass attached to the inner side of the host tube (Fig. 6H).

The component communities differed also in the relative abundance of a particular species. In the community associated with *C*. cf. *appendiculatus*, *O. pettiboneae* was dominant in terms of both prevalence and abundance. In the community associated with *Chaetopterus* sp., the dominance of the most abundant symbiont, *P*. cf. *heok* is less distinctive, with the role that other species had in the community structure being more relevant (Fig. 7C).

The average length of infested and non-infested tubes of *C*. *appediculatus* does not differ significantly (41.2–42.3, *t*-test, *p* = 0.65). The number of both symbiotic species and individuals do not show any significant correlation with host tube length in both chaetopterid species.

## Discussion

### Community dissimilarity

Our results demonstrate a strict segregation in species composition of the communities associated with the two Vietnamese syntopic species of *Chaetopterus*, which had no species in common. However, at higher taxonomic levels (i.e., family, order and class), they were similar to each other and also resembled the symbiotic communities associated with other species of *Chaetopterus* and, even, echiuran worms in harbouring scale-worms, pocellanid and pinnotherid decapods and fishes (Anker et al., 2005; Gray, 1961; Ng & Sasekumar, 1993; Petersen & Britayev, 1997).

The two Vietnamese species of *Chaetopterus* are very similar in body morphology and tube shape, as well as in their trophic-functional characteristics. Thus, no reasons linked to host morphology were evident allowing us to explain the dissimilarity in symbiotic species composition. We suggest therefore that community composition appears to be determined by historical events rather than by the physical or biological habitat characteristics. We may hypothesize that both host species were originally allopatric. Thus, the evolutionary establishment of the respective specialized symbiotic associations would likely occur in different, separated geographical areas, with the posterior disappearance of geographical barriers leading to the current overlapping distribution. Once established, the respective symbiotic communities would be maintained by interspecific competition, leading to symbiont specialization to their respective host species as well as to preventing the exchange of symbionts between hosts when becoming sympatric, even being as closely related as is the case for these two species of *Chaetopterus*. However, our hypothesis does not exclude the possible existence of differences in host physiology or behaviour that would enhance the ability of the specialized symbionts to compete with possible invaders, thus contributing to maintain the differences in community composition.

Further assessment of this hypothesis would require an experimental approach to analyse the possible existence of a host-factor allowing the respective symbiont to recognize their own hosts, as well as to check the ability of the symbionts from one host to infest the other. In parallel, the regularity of the community segregation would have to be checked by more extensive field sampling addressed to discard (or reveal) the presence of additional symbionts on the alternative host species. This is particularly relevant for *Chaetopterus* sp. whose accumulation curve (Fig. 7A) supports an expected increase in the respective number of symbiotic species with sampling size. An additional, but not less pertinent question would be to assess the degree of specialization of the symbionts found in the two species of *Chaetopterus*, either based on previously published data or on our own observations. Therefore, it would be particularly relevant to consider whether they are obligatory or facultative and, in the case of obligatory symbionts, to further assess their degree of specificity (which may range from species-specific to opportunistic).

### Symbionts’ specialization

Among the seven species of macroinvertebrates associated with *C*. cf. *appendiculatus* and *Chaetopterus* sp., four (i.e., one polychaete *O. pettiboneae*, one crab, *E. cometes*, and one fish, *O. fowleri*) are known as obligatory symbionts of chaetopterids and other benthic organisms. *Onuxodon fowleri* also lives in the mantle cavity of bivalves and inside holothurians (Markle & Olney, 1990; Parmentier, Chardon & Vanderwalle, 2002). In our samples, five of six individuals were juveniles, which allows us to suggest that they are employing *C*. cf. *appendiculatus* as temporal or intermediate hosts. The porcellanid *E. cometes* was reported from shallow waters off Singapore, living in association with a species of *Chaetopterus*, identified as *C. variopedatus* but this identification is undoubtedly incorrect (Ng & Nakasone, 1993). In turn, the scale-worm *O. pettiboneae* was first reported from Ambon Island (Indonesia) and later from the coasts of Vietnam, always in association with *C*. cf. *appendiculatus* (Britayev & Martin, 2005; Petersen & Britayev, 1997; this paper).

The four other species appear to be undescribed and are now being analysed by the corresponding specialists. However, we may infer some considerations on their degree of specialization based on existing papers dealing with the ecology and morphology of some closely related taxa. Concerning the symbiotic crabs, the porcellanid *Polyonyx* sp. belongs to the “*Polyonyx sinensis*” species complex, usually obligatory associates of tubicolous polychaetes, mainly with species of *Chaetopterus* (B Werding, 2016, unpublished data), while *Polyonyx* cf. *heok* belongs to the “*Polyonyx pedalis*” complex and the same or a very similar species has been recently reported from Singapore living in association with *Chaetopterus* cf. *pacificus* (Osawa & Ng, 2016). This suggests that both porcellanids are obligate and, probably, specialized symbionts of *Chaetopterus* sp. In turn, *Tetrias* sp. belongs to the Pinnotheridae, a family that mainly includes symbiotic species living as endo- or ectosymbionts in mollusc mantle cavities, polychaete burrows, echinoid integuments or tunicate branchial sacs (Drake et al., 2014). Among them, two species of *Tetrias* are currently known. *Tetrias fischerii* (Milne-Edwards, 1867) has been reported as symbiont of bivalves and annelids, while the host of *Tetrias scabripes* Rathbun, 1898 is unknown (Schmitt, McCain & Davidson, 1973). Although this cannot be assessed from our data, we suggest that the species associated with the Vietnamese *Chaetopterus* sp. is a specialized obligatory symbiont.

The third undescribed species, the nudibranch *Phestilla* sp., has several behavioural (i.e., two or more individuals sharing the same host, egg-masses attached to the inner tube surface) and morphological (i.e., posterior end working as a sucker, overall body shape mimicking that of the host) features clearly pointing toward a specialized symbiotic mode of life. This lead us to consider the species as the first know nudibranch living as a symbiont with a marine annelid host. Nudibranchs are well known as aposematic or mimetic organisms (Edmunds, 1987; Gosliner & Behrens, 1989; Rudman, 1991), some of them being considered as true symbionts. Among them, there are some species of *Phestilla*, which lives in association with corals and are highly specialized predators (Faucci, Toonen & Hadfield, 2007; Robertson, 1970), while the exact nature of the association of the Vietnamese *Phestilla* sp. and *Chaetopterus* sp. would need further research to be defined. The single related association occurred between the goniodorid nudibranch *Lophodoris scala* Marcus & Marcus, 1970 and the innkeeper echiurid *Lissomyema exilii* (Muüller, 1883). In this case, the nudibranch lives inside the host burrow, sometimes creeping along the host trunk and feeding, possibly exclusively, on *Loxosomella* spp., an entoproct that colonizes the burrow walls (Ditadi, 1982; Marcus & Marcus, 1970), which seems not to be the case for the Vietnamese species.

Accordingly, all symbionts found in association with *Chaetopterus* sp. and *C*. cf. *appendiculatus* have to be considered as obligatory symbionts. Among them, the less specialized is *O. fowleri*, which is known to infest hosts belonging to different types of animals (molluscs and polychaetes). The porcellanid crabs *E. cometes* and *P*. cf. *heok* are probably genus-specific symbionts, while the scale-worm *O. pettiboneae*, together with the other porcellanid crab *Polyonyx* sp. and the tergipedid nudibranch *Phestilla* sp., must be considered as species-specific symbionts. The specificity of the pinnotherid crab *Tetrias* sp. is not clear at this time. However, taking into account the relative abundance of pinnotherids among symbionts of *Chaetopterus* species (Petersen & Britayev, 1997; Schmitt, McCain & Davidson, 1973), we could also propose that it should be considered as a specialized symbiont, at least at family level.

Therefore, we consider all symbionts found in association with the two Vietnamese species of *Chaetopterus* as being, or tending to be, specialized symbionts, the single exception being the carapid fish.

### Possible causes of observed differences in community structure

We found substantial differences in the structure of the symbiotic communities associated with *Chaetopterus* sp. and *C*. cf. *appendiculatus*. The first shows a significantly higher species richness and mean abundance than the second, while the second was clearly dominated by the presence of a single species, both in terms of abundance and frequency (Table 6, Fig. 6C). Taking into account that body size and tube length of *Chaetopterus* sp. are significantly lower than those of *C*. cf. *appendiculatus*, this situation is particularly unexpected. Usually, species richness and abundance increase with the increasing host size (e.g., Abele & Patton, 1976; Ribeiro, Omena & Muricy, 2003). Thus, the sitiuation of the Vietnamese partnership requires specific considerations.

We suggest that several factors are shaping the differences in the structure of the symbiotic communities associated with *Chaetopterus* sp. and *C*. cf. *appendiculatus.* Despite both host species having low population densities in Nhatrang Bay, that of *Chaetopterus* sp. was significantly lower, which would likely force the associated symbionts to use (and share) the few available hosts. This would possible explain the higher number of species in its associated community, as well as the fact that all host individuals of *Chaetopterus* sp. found in Nhatrang Bay harboured symbionts, in contrast to *C. appenciculatus* whose maximum prevalence was around 70%. Alternatively, the low density of both host populations may impede the secondary dispersion of the symbionts, which has been considered as a key mechanisms shaping the establishment and functioning of marine symbiotic communities (Mekhova et al., 2015) and raises the question on the adults’ ability of long-distance migration.

Based on species and individual’s distributions among hosts, we also hypothesized that another factor determining the observed differences in symbiotic community structure could be the existence of inter- and intraspecific competition. In fact, most tubes of *Chaetopterus* sp. were occupied by a minimum of two symbiotic species, this co-occurence being very rare in *C*. cf. *appendiculatus*, where each host individual was infested by one symbiotic species (Fig. 7B). The single exception was a host tube shared by *O. pettiboneae* and *E. cometes*. Accordingly, we suggest that the main driving factors may be resource partitioning between symbiotic species having different sizes, in the case of *Chaetopterus* sp., and strong interspecific interactions, in the case of *C*. cf. *appendiculatus* as previously reported for holothurian hosts (Lyskin & Britayev, 2005).

The characteristics of the symbiotic community structure associated with *C*. cf. *appendiculatus*, in which one host was usually occupied by one symbiotic species (Table 5), suggest the existence of interspecific competition between the polychaete and fish symbionts. In turn, the fact that there was a single symbiont per host (Table  5) supports the existence of intraspecific competition among polychaetes and fish individuals, respectively. At least for the polychaete, this hypothesis was supported by our direct observations in experimental aquaria, where individuals of *O. pettiboneae* were found to fight when trying to occupy the same host tube, as well as by the high frequency of body traumas present (TA Britayev & D Martin, 2016, unpublished data). In contrast, the bulk of *Chaetopterus* sp. symbionts were crabs (Fig. 7C). Territorial defence is a well-known phenomenon in symbiotic decapods too (Baeza, Stotz & Thiel, 2002; Huber, 1987; Vannini, 1985). However, their behaviour has a sexual component, as they often form heterosexual pairs consisting of gravid males and females co-inhabiting the same host (Castro, 2015; Patton, 1994), which was exactly the case of most porcellanid and pinnotherid crabs inhabiting the tubes of the two Vietnamese species of *Chaetopterus* (Table 5). This behaviour lead to a significant increase in the abundance of symbiotic individuals in the community associated with *Chaetopterus* sp. Therefore, while interspecific interactions seemed to affect both species richness and abundance, the intraspecific ones only affected the abundance.

Our observations support two main factors determining the structure of symbiotic communities associated with *Chaetopterus* sp. and C. cf. *appendiculatus* in Nhatrang Bay: the density of host populations and competition (both inter- and intraspecific). Moreover, the observed differences in community structure appear to be strictly related to the respective species composition. Accordingly, living in pairs and resource partitioning led to species coexisting in the tubes of *Chaetopterus* sp. and establish a tightly packed component community showing high species richness and mean intensity, together with a low species dominance. In contrast, the aggressive, strictly territorial species associated with *C*. cf. *appendiculatus* established a component community strongly dominated by host being inhabited by a single species and, thus, low species richness and mean intensity.

The existence of two closely related host species with overlapping distributions but harbouring very different symbiotic communities seems to be unusual. We suggest that it may probably be related with the scarcity of data currently available on the structure of symbiotic communities in marine environments. However, the situation is certainly not unique, as at least an additional example has been recently reported from Nhatrang Bay. In this case, the hosts were comatulid crinoids *Comanthus gisleni* Rowe, Hoggett, Birtles & Vail, 1986 and *C. parvicirrus* (Müller, 1841) (Mekhova & Britayev, 2012). Consequently, we expect further worldwide studies to discover more syntopic hosts harbouring symbiotic communities with contrasted composition and structure.

## Conclusions

Two symbiotic communities inhabit the morphologically similar and syntopic species of the tube-dwelling chaetopterid polychaetes *Chaetopterus* sp. and *C*.cf. *appendiculatus* in Nhatrang Bay. They are mostly composed of specifically specialized species and show a very different composition. The current situation has been attributed to an initially allopatric host distribution allowing the symbiotic communities to be established independently. This is then followed by the subsequent disappearance of the original geographical barriers leading to the current sympatry. The present symbiotic communities differ in structural characteristics (i.e., species richness, mean intensity and species dominance) as a consequence of the differences in host density but also of the existing intra- and interspecific interactions that, in turn, depends on the behaviour of the respective symbiotic species. Mating pairs and partitioned resources lead to a high diversity and intensity in the community associated with *Chaetopterus* sp., while the aggressive and territorial species associated with *C*. cf. *appendiculatus* led to a community with low diversity and intensity but with a strong dominance of a single species.

The hypotheses postulating a similar composition for the symbiotic communities established on closely related hosts seems to reflect a rather common situation in marine ecosystems and, certainly, our results do not allow us to reject it. In fact, the opposite situation was observed within our data, with two taxonomically related hosts living in the same habitat that harbour symbiotic communities with contrasted species composition. Therefore, we hypothesize on the possible reasons explaining their establishment. We also highlight that the situation of the Vietnamese partnerships is certainly not unique and should be considered as an interesting model to further assess different evolutionary and ecological aspects of the establishment of a symbiotic community.

Our results also highlight the importance of studying previously unknown symbiotic associations, which may provide key information allowing the complex network of relationships driving the functioning of the marine ecosystems, particularly in benthic environments, to be understood. Moreover, they are crucial in revealing the hidden biodiversity of the oceans, as supported by the fact that at least five of the nine species herein studied are currently undescribed.

##  Supplemental Information

10.7717/peerj.2930/supp-1 Table S1 Main data on the two *Chaetopterus* hosts Main data on the two *Chaetopterus* hosts (tube length in cm, body volume in ml), symbionts’ abundance and number of species, and number of specimens of each symbiotic species found in each individual host specimen tube.

10.7717/peerj.2930/supp-2 Table S2 Body volume and tube length of *Chaetopterus* cf. *appendiculatus* Body volume (ml) and tube length (cm) of the 45 specimens of *Chaetopterus* cf. *appendiculatus* selected to estimate the relationship between these two measures in the analysis of community structure.

## Additional Information and Declarations

### Field Study Permissions

The following information was supplied relating to field study approvals (i.e., approving body and any reference numbers): Russian–Vietnamese Tropical Center approved the collection of the samples and animals used in our article.

### Data Availability

The following information was supplied regarding data availability: ResearchGate: https://www.researchgate.net/publication/310159685_Phestilla_sp.

## References

[ref-1] Abele LG, Patton WK (1976). The size of coral heads and the community biology of associated decapod crustaceans. Journal of Biogeography.

[ref-2] Anker A, Murina GV, Lira C, Vera Caripe JA, Palmer AR, Jeng MS (2005). Macrofauna associated with echiuran burrows: a review with new observations of the innkeeper worm, *Ochetostoma erythrogrammon* Leuckart and Rüppel, in Venezuela. Zoological Studies.

[ref-3] Antokhina TI, Savinkin OV, Britayev TA (2012). Asteroidea of Vietnam with some notes on their symbionts. Benthic fauna of the Bay of Nhatrang, Southern Vietnam, Vol 2.

[ref-4] Baeza J, Stotz W, Thiel M (2002). Agonistic behaviour and development of territoriality during ontogeny of the sea anemone dwelling crab *Allopetrolisthes spinifrons* (H. Milne Edwards, 1837) (Decapoda: Anomura: Porcellanidae). Marine and Freshwater Behaviour and Physiology.

[ref-5] Baeza JA, Thiel M, Watling L (2015). Crustaceans as symbionts: an overview of their diversity, host use, and lifestyles. Lifestyles and Feeding Biology.

[ref-6] Barel CDN, Kramers PGN (1977). A survey of the echinoderm associates of the North-East Atlantic area. Zoologische Verhandelingen Leiden.

[ref-7] Bhaud M (1998). The spreading potential of polychaete larvae does not predict adult distributions, consequences for conditions of recruitment. *Hydrobiologia*.

[ref-8] Britayev TA (1993). *Pilargis berkeleyae* (Polychaeta, Pilargidae) as a commensal of a sedentary polychaete *Chaetopterus cautus* (Chaetopteridae). Zoologicheski Zhurnal.

[ref-9] Britayev TA, Martin D (2005). Scale-worms (Polychaeta, Polynoidae) associated with chaetopterid worms (Polychaeta, Chaetopteridae), with description of a new genus and species. Journal of Natural History.

[ref-10] Britayev TA, Martin D (2016). Chaetopteridae Audouin & Milne Edwards, 1833. Handbook of zoology annelida.

[ref-11] Carr CM, Hardy SM, Brown TM, Macdonald TA, Hebert PD (2011). A tri-oceanic perspective: DNA barcoding reveals geographic structure and cryptic diversity in Canadian polychaetes. PLOS ONE.

[ref-12] Castro P, Castro P, Davie P,  Guinot D, Schram FR, Von Vaupel Klein JC (2015). Symbiotic Brachyura. Treatise on Zoology-Anatomy, Taxonomy, Biology The Crustacea, Volume 9 Part C (2 vols).

[ref-13] Claparéde É Les Annélides Chétopodes du Golte de Naples. Seconde partie. Annélides sédentaires. Mémoires de la Société de Physique et D’histoire Naturelle de Genève.

[ref-14] Cuvier G (1830). Le règne animal distribué d’après son organisation, pour servir de base a l’histoire naturelle des animaux et d’introduction a l’anatomie comparée.

[ref-15] Deheyn D, Lyskin SA, Eeckahaut I (2006). Assemblages of symbionts in tropical shallow-water crinoids and assessment of symbionts’ host-specificity. Symbiosis.

[ref-16] Ditadi ASF (1982). On the burrows of echiuran worms (Echiura): a survey. Boletim de Zoologia da Universidade de Sao Paulo.

[ref-17] Drake P, Marco-Herrero H, Subida MD, Arias AM, Cuesta JA (2014). Host use pattern of the pea crab *Afropinnotheres monodi*: potential effects on its reproductive success and geographical expansion. Marine Ecology Progress Series.

[ref-18] Duffy JE, Kikuchi T (2002). The ecology and evolution of eusociality in sponge-dwelling shrimp. Genes, behaviors and evolution of social insects.

[ref-19] Edmunds M (1987). Color in opisthobranchs. American Malacological Bulletin.

[ref-20] Enders HE (1909). A study of the life-history and habits of *Chaetopterus variopedatus*. Journal of Morphology.

[ref-21] Faucci A, Toonen RJ, Hadfield MG (2007). Host shift and speciation in a coral-feeding nudibranch. Proceedings of the Royal Society of London B: Biological Sciences.

[ref-22] Felsenstein J (1985). Confidence limits on phylogenies: an approach using the bootstrap. Evolution.

[ref-23] Gosliner TM, Behrens DW, Wicksten M (1989). Special resemblance, aposematic coloration and mimicry in opisthobranch gastropods.

[ref-24] Goto R, Kato M (2011). Geographic mosaic of mutually exclusive dominance of obligate commensals in symbiotic communities associated with a burrowing echiuran worm. Marine Biology.

[ref-25] Gray IE (1961). Changes in abundance of the commensal crabs of *Chaetopterus*. The Biological Bulletin.

[ref-26] Grube AE (1874). Descriptiones Annulatorum novorum mare Ceylonicum habitantium ab honoratissimo Holdsworth collectorum. Proceedings of the Zoological Society of London.

[ref-27] Hartman O (1959). Catalogue of the polychaetous Annelids of the World, parts 1–2. Allan Hancock Foundation Publications, Occasional Papers.

[ref-28] Hoeksema BW, Van der Meij SE, Fransen CH (2012). The mushroom coral as a habitat. Journal of the Marine Biological Association of the United Kingdom.

[ref-29] Huber ME (1987). Aggressive behaviour of *Trapezia intermedia* Miers and *T. digitalis* Latreilli (Brachiura: Xantidae). Journal of Crustacean Biology.

[ref-30] Kumar S, Stecher G, Tamura K (2016). MEGA7: molecular evolutionary genetics analysis version 7.0 for bigger datasets. Molecular Biology and Evolution.

[ref-31] Lyskin SA, Britayev TA (2005). Symbionts of holothurians from South Vietnam: intra- and interspecific interactions. Doklady Biological Sciences.

[ref-32] Marcus E, Marcus EDB-R (1970). Opisthobranchs from Curaçao and faunistically related regions. Studies on the Fauna of Curaçao and other Caribbean Islands.

[ref-33] Marenzeller E (1879). Südjapanische Anneliden. I. Amphinomea, Aphroditea, Lycoridea, Phyllodocea, Hesionea, Syllidea, Eunicea, Glycerea, Sternaspidea, Chaetopterea, Cirratulea, Amphictenea. *Denkschriften der Mathematisch—Naturwissenschaftlichen Classe der Kaiserlichen Akademie der Wissenschaften*.

[ref-34] Markle DF, Olney JE (1990). Systematics of the pearlfishes (Pisces: Carapidae). Bulletin of Marine Science.

[ref-35] Martin D, Gil J, Carreras-Carbonell J, Bhaud M (2008). Description of a new species of *Mesochaetopterus* (Annelida, Polychaeta, Chaetopteridae), with re-description of *Mesochaetopterus xerecus* and an approach to the phylogeny of the family. Zoological Journal of the Linnean Society.

[ref-36] Mekhova ES, Britayev TA, Britayev TA, Pavlov DS (2012). Feather stars (Crinoidea, Comatulida) of Nhatrang Bay, Vietnam: fauna, habitat and symbionts. Benthic fauna of the Bay of Nhatrang, Southern Vietnam.

[ref-37] Mekhova ES, Dgebuadze PY, Mikheev VN, Britayev TA (2015). Colonization of depopulated crinoids by symbionts: who comes from the bottom and who from the water column?. Journal of the Marine Biological Association of the United Kingdom.

[ref-38] Milne-Edwards A (1867). Descriptions de quelques especes nouvelles de Crustaces Brachyures. Annales de la Socieéé entomologique de France.

[ref-39] Muller F (1883). Über einiger neue *Thalassema*. Zeitschri für wissenscha liche Zoologie.

[ref-40] Ng P, Nakasone Y (1993). Taxonomy and ecology of the porcellanid crab *Polyonyx cometes* Walker, 1887 (Crustacea: Decapoda), with description of a new genus. Journal of Natural History.

[ref-41] Ng PKL, Sasekumar A (1993). A new species of *Polyonyx* Stimpson, 1858, of the *P. sinensis* group (Crustacea: Decapoda: Anomura: Porcellanidae) commensal with a chaetopterid worm from Peninsular Malaysia. Zoologische Mededelingen.

[ref-42] Nishi E (2001). Partial revision of Japanese *Chaetopterus* (Chaetopteridae, Polychaeta), including description of three new species from southern Pacific side of central Japan. Actinia Bulletin of Manazuru Marine Laboratory for Science Education Faculty of Education and Human Sciences, Yokohama National University.

[ref-43] Nishi E, Arai H, Sasanuma S-I (2000). A new species of *Chaetopterus* (Polychaeta: Chaetopteridae) from off Tokyo Bay, Central Japan, with comments on its Bioluminescence. Actinia Bulletin of Manazuru Marine Laboratory for Science Education Faculty of Education and Human Sciences, Yokohama National University.

[ref-44] Nishi E, Hickman Jr CP, Bailey-Brock J (2009). *Chaetopterus* and *Mesochaetopterus* (Polychaeta: Chaetopteridae) rom the Galapagos Islands, with descriptions of four new species. Proceedings of the Academy of Natural Sciences of Philadelphia.

[ref-45] Osawa M, Ng PK (2016). Revision of *Polyonyx pedalis* Nobili, 1906 (Crustacea: Decapoda: Anomura: Porcellanidae), with descriptions of three new species. Raffles Bulletin of Zoology, Supplement.

[ref-46] Osborn KJ, Rouse GW, Goffredi SK, Robison BH (2007). Description and relationships of *Chaetopterus pugaporcinus*, an unusual pelagic polychaete (Annelida, Chaetopteridae). The Biological Bulletin.

[ref-47] Parmentier E, Chardon M, Vanderwalle P, Aerts P, D’Août K, Herrel K, Van Damme R (2002). Preliminary study on the ecomorphological signification of the sound-producing complex in Carapidae. Topics in Functional and Ecological Vertebrate Morphology.

[ref-48] Patton WK (1994). Distribution and ecology of animals associated with branching corals (*Acropora* spp.) from the Great Barrier Reef, Australia. Bulletin of Marine Science.

[ref-49] Petersen ME (1984a). *Chaetopterus variopedatus* (Annelida, Polychaeta): another victim of the “characteristic species” disease. American Zoologist.

[ref-50] Petersen ME (1984b). *Chaetopterus variopedatus* (Renier) (Annelida: Polychaeta: Chaetopteridae): a species complex. What species are being used at MBL?. Biological Bulletin Marine Biological Laboratory, Woods Hole.

[ref-51] Petersen ME (1997). Contribution to a revision of *Chaetopterus* Cuvier (Polychaeta: Chaetopteridae): redescription of *C. appendiculatus* Grube and *C. cautus* Marenzeller, with comments on some other species. Bulletin of Marine Science.

[ref-52] Petersen ME, Britayev TA (1997). A new genus and species of polynoid scaleworm commensal with *Chaetopterus appendiculatus* Grube from the Banda Sea (Annelida: Polychaeta), with a review of commensals of Chaetopteridae. Bulletin of Marine Science.

[ref-53] Pleijel F, Jondelius U, Norlinder E, Nygren A, Oxelman B, Schander C, Sundberg P, Thollesson M (2008). Phylogenies without roots? A plea for the use of vouchers in molecular phylogenetic studies. Molecular Phylogenetics and Evolution.

[ref-54] Poulin R (1998). Large-scale patterns of host use by parasites of freshwater fishes. Ecology Letters.

[ref-55] Rathbun MJ (1898). The Brachyura of the biological expedition to the Florida Keys and the Bahamas in 1893. Bulletin from the Laboratories of Natural History of the State University of Iowa.

[ref-56] Renier SA, Meneghini G (1804). Prospetto della Classe dei Vermi, nominati el ordinati secondo il Sistema de Bosc. Osservazioni postume di Zoologia Adriatica del Professore Stefano Andrea Renier, membro effettivo dell’ Istituto Italiano.

[ref-57] Ribeiro SM, Omena EP, Muricy G (2003). Macrofauna associated to *Mycale microsigmatosa* (Porifera, Demospongiae) in Rio de Janeiro State, SE Brazil. Estuarine, Coastal and Shelf Science.

[ref-58] Robertson R (1970). Review of the predators and parasites of stony corals, with special reference to symbiotic prosobranch gastropods. Pacific Science.

[ref-59] Rudman WB (1991). Purpose in pattern: the evolution of colour in chromodorid nudibranchs. Journal of Molluscan Studies.

[ref-60] Saitou N, Nei M (1987). The neighbor-joining method: a new method for reconstructing phylogenetic trees. Molecular Biology and Evolution.

[ref-61] Schmitt WL, McCain JC, Davidson ES, Gruner H-E, Holthuis LB (1973). Family Pinnotheridae. Decapoda I. Brachyura I. Crustaceorum catalogus.

[ref-62] Smith CL (1964). Some pearlfishes from Guam, with notes on their ecology. Pacific Science.

[ref-63] Stella J, Jones G, Pratchett M (2010). Variation in the structure of epifaunal invertebrate assemblages among coral hosts. Coral Reefs.

[ref-64] Sun Y, Qiu JW (2014). A new species of *Chaetopterus* (Annelida, Chaetopteridae) from Hong Kong. Memoirs of Museum Victoria.

[ref-65] Tamura K, Nei M, Kumar S (2004). Prospects for inferring very large phylogenies by using the neighbor-joining method. Proceedings of the National Academy of Sciences of the United States of America.

[ref-66] Thiel M, Baeza JA (2001). Factors affecting the social behaviour of crustaceans living symbiotically with other marine invertebrates: a modelling approach. Symbiosis.

[ref-67] Vannini M (1985). A shrimp that speaks crab-ese. Journal of Crustacean Biology.

[ref-68] Walker AO (1887). Notes on a collection of Crustacea from Singapore. Journal of the Linnean Society of London, Zoology.

[ref-69] Williams JD, McDermott JJ (2004). Hermit crab biocoenoses: a worldwide review of the diversity and natural history of hermit crab associates. Journal of Experimental Marine Biology and Ecology.

