# Supplementary material for: Do syntopic host species harbour similar symbiotic communities? The case of Chaetopterus spp. (Annelida: Chaetopteridae)
Source: PeerJ. 2017 Feb 2;5:e2930. doi: 10.7717/peerj.2930 (PMC5292031; doi:10.7717/peerj.2930)
Supplement: Table S1 — Main data on the two Chaetopterus hosts (tube length in cm, body volume in ml), symbionts’ abundance and number of species, and number of specimens of each symbiotic species found in each individual host specimen tube. [file peerj-05-2930-s001.docx]

|  | Host |  |  | Symbiont |  |  | Polychaeta |  | Fish |  | Decapoda |  |  |  |  | Nudibranch |
| --- | --- | --- | --- | --- | --- | --- | --- | --- | --- | --- | --- | --- | --- | --- | --- | --- |
| Specimen | Tube length | Body volume |  | abudance | N of species |  | *Ophthalmonoe pettiboneae* |  | *Onuxodon fowleri* |  | *Eulenaios cometes* | *Polyonyx* cf. *heox* | *Polyonyx* sp. | *Tetrias* sp. |  | Tergipedidae gen.sp. |
| *Chaetopterus sp.* | |  |  |  |  |  |  |  |  |  |  |  |  |  |  |  |
| 4 | 47 | N/A |  | 4 | 2 |  | - |  | - |  | - | 2 | 2 | - |  | - |
| 7 | 46 | N/A |  | 3 | 2 |  | - |  | - |  | - | 2 | 1 | - |  | - |
| 16 | 37 | 11 |  | 4 | 2 |  | - |  | - |  | - | 2 | - | - |  | 2 |
| 22 | 50 | 32 |  | 3 | 2 |  | - |  | - |  | - | 2 | 1 | - |  | - |
| 46 | 58 | 25 |  | 4 | 2 |  | - |  | - |  | - | 2 | 2 | - |  | - |
| 66 | 35 | 4 |  | 3 | 2 |  | - |  | - |  | - | - | 1 | 2 |  | - |
| 81 | N/A | 16 |  | 2 | 2 |  | - |  | - |  | - | 1 | 1 | - |  | - |
| 82 | 52 | 17 |  | 2 | 2 |  | - |  | - |  | - | 1 | - | - |  | 1 |
| 88 | 51 |  |  | 1 | 1 |  | - |  | - |  | - | 1 | - | - |  | - |
| 93 | 49 | 19 |  | 2 | 1 |  | - |  | - |  | - | 2 | - | - |  | - |
| 94 | 23 | 2 |  | 5 | 2 |  | - |  | - |  | - | 2 | - | - |  | 3 |
| *Chaetopterus* cf. *appendiculatus* | | |  |  |  |  |  |  |  |  |  |  |  |  |  |  |
| 1 | 64 | 33 |  | 3 | 2 |  | 1 |  | - |  | 2 | - | - | - |  | - |
| 2 | 66 | N/A |  | 1 | 1 |  | 1 |  | - |  | - | - | - | - |  | - |
| 3 | 65 | N/A |  | 1 | 1 |  | 1 |  | - |  | - | - | - | - |  | - |
| 5 | 88 | N/A |  | 1 | 1 |  | 1 |  | - |  | - | - | - | - |  | - |
| 6 | 44 | 32 |  | 1 | 1 |  | 1 |  | - |  | - | - | - | - |  | - |
| 8 | 61 | 44 |  | 1 | 1 |  | 1 |  | - |  | - | - | - | - |  | - |
| 9 | 47 | 28 |  | 1 | 1 |  | 1 |  | - |  | - | - | - | - |  | - |
| 10 | 71 | 49 |  | 1 | 1 |  | 1 |  | - |  | - | - | - | - |  | - |
| 11 | 77 | 44 |  | 1 | 1 |  | 1 |  | - |  | - | - | - | - |  | - |
| 12 | 70 | 44 |  | 1 | 1 |  | 1 |  | - |  | - | - | - | - |  | - |
| 13 | 66 | 31 |  | 1 | 1 |  | 1 |  | - |  | - | - | - | - |  | - |
| 14 | 67 | 44 |  | 1 | 1 |  | 1 |  | - |  | - | - | - | - |  | - |
| 15 | 62 | 46 |  | 1 | 1 |  | 1 |  | - |  | - | - | - | - |  | - |
| 17 | 60 | 37 |  | 1 | 1 |  | 1 |  | - |  | - | - | - | - |  | - |
| 18 | 59 | 40 |  | 1 | 1 |  | 1 |  | - |  | - | - | - | - |  | - |
| 19 | 69 | 45 |  | 1 | 1 |  | 1 |  | - |  | - | - | - | - |  | - |
| 20 | 71 | 34 |  | 1 | 1 |  | 1 |  | - |  | - | - | - | - |  | - |
| 21 | 67 | 41 |  | 1 | 1 |  | 1 |  | - |  | - | - | - | - |  | - |
| 23 | 61 | 34 |  | 1 | 1 |  | 1 |  | - |  | - | - | - | - |  | - |
| 24 | 58 | N/A |  | 1 | 1 |  | 1 |  | - |  | - | - | - | - |  | - |
| 25 | 74 | 48 |  | 1 | 1 |  | 1 |  | - |  | - | - | - | - |  | - |
| 26 | 69 | 43 |  | 1 | 1 |  | 1 |  | - |  | - | - | - | - |  | - |
| 27 | 70 | 46 |  | 1 | 1 |  | 1 |  | - |  | - | - | - | - |  | - |
| 28 | 68 | 72 |  | 1 | 1 |  | 1 |  | - |  | - | - | - | - |  | - |
| 29 | 62 | 40 |  | 1 | 1 |  | 1 |  | - |  | - | - | - | - |  | - |
| 30 | 64 | 49 |  | 1 | 1 |  | 1 |  | - |  | - | - | - | - |  | - |
| 31 | 75 | 36 |  | 1 | 1 |  | 1 |  | - |  | - | - | - | - |  | - |
| 32 | 61 | 32 |  | 1 | 1 |  | 1 |  | - |  | - | - | - | - |  | - |
| 33 | 75 | 48 |  | 1 | 1 |  | 1 |  | - |  | - | - | - | - |  | - |
| 34 | 52 | 25 |  | 1 | 1 |  | 1 |  | - |  | - | - | - | - |  | - |
| 35 | N/A | 38 |  | 1 | 1 |  | 1 |  | - |  | - | - | - | - |  | - |
| 36 | 59 | 24 |  | 1 | 1 |  | 1 |  | - |  | - | - | - | - |  | - |
| 37 | 61 | 43 |  | 1 | 1 |  | 1 |  | - |  | - | - | - | - |  | - |
| 38 | 68 | N/A |  | 1 | 1 |  | 1 |  | - |  | - | - | - | - |  | - |
| 39 | 72 | 50 |  | 1 | 1 |  | 1 |  | - |  | - | - | - | - |  | - |
| 40 | 59 | 32 |  | 1 | 1 |  | 1 |  | - |  | - | - | - | - |  | - |
| 41 | 58 | 28 |  | 1 | 1 |  | 1 |  | - |  | - | - | - | - |  | - |
| 42 | 65 | 40 |  | 1 | 1 |  | 1 |  | - |  | - | - | - | - |  | - |
| 43 | 64 | 50 |  | 1 | 1 |  | 1 |  | - |  | - | - | - | - |  | - |
| 44 | 71 | 49 |  | 1 | 1 |  | 1 |  | - |  | - | - | - | - |  | - |
| 45 | 79 | 62 |  | 1 | 1 |  | 1 |  | - |  | - | - | - | - |  | - |
| 47 | 66 | 48 |  | 1 | 1 |  | 1 |  | - |  | - | - | - | - |  | - |
| 48 | 76 | 51 |  | 1 | 1 |  | 1 |  | - |  | - | - | - | - |  | - |
| 49 | 72 | 51 |  | 1 | 1 |  | 1 |  | - |  | - | - | - | - |  | - |
| 50 | 64 | 45 |  | 1 | 1 |  | 1 |  | - |  | - | - | - | - |  | - |
| 51 | 56 | 41 |  | 1 | 1 |  | 1 |  | - |  | - | - | - | - |  | - |
| 52 | 55 | 49 |  | 1 | 1 |  | 1 |  | - |  | - | - | - | - |  | - |
| 53 | 63 | 52 |  | 1 | 1 |  | 1 |  | - |  | - | - | - | - |  | - |
| 54 | 70 | 44 |  | 1 | 1 |  | 1 |  | - |  | - | - | - | - |  | - |
| 55 | 69 | 45 |  | 1 | 1 |  | 1 |  | - |  | - | - | - | - |  | - |
| 56 | 56 | 39 |  | 1 | 1 |  | 1 |  | - |  | - | - | - | - |  | - |
| 57 | 74 | 47 |  | 1 | 1 |  | - |  | 1 |  | - | - | - | - |  | - |
| 58 | 59 | 39 |  | 1 | 1 |  | - |  | 1 |  | - | - | - | - |  | - |
| 59 | 48 | 39 |  | 1 | 1 |  | - |  | 1 |  | - | - | - | - |  | - |
| 60 | 67 | 56 |  | 1 | 1 |  | - |  | 1 |  | - | - | - | - |  | - |
| 61 | 50 | 23 |  | 1 | 1 |  | - |  | 1 |  | - | - | - | - |  | - |
| 62 | 66 | N/A |  | - | - |  | - |  | - |  | - | - | - | - |  | - |
| 63 | 76 | N/A |  | - | - |  | - |  | - |  | - | - | - | - |  | - |
| 64 | N/A | N/A |  | - | - |  | - |  | - |  | - | - | - | - |  | - |
| 65 | 61 | N/A |  | - | - |  | - |  | - |  | - | - | - | - |  | - |
| 67 | 77 | N/A |  | - | - |  | - |  | - |  | - | - | - | - |  | - |
| 68 | 60 | 31 |  | - | - |  | - |  | - |  | - | - | - | - |  | - |
| 69 | 67 | 50 |  | - | - |  | - |  | - |  | - | - | - | - |  | - |
| 70 | 50 | N/A |  | - | - |  | - |  | - |  | - | - | - | - |  | - |
| 71 | N/A | 36 |  | - | - |  | - |  | - |  | - | - | - | - |  | - |
| 72 | 76 | 54 |  | - | - |  | - |  | - |  | - | - | - | - |  | - |
| 73 | 68 | 45 |  | - | - |  | - |  | - |  | - | - | - | - |  | - |
| 74 | 78 | 57 |  | - | - |  | - |  | - |  | - | - | - | - |  | - |
| 75 | 66 | 40 |  | - | - |  | - |  | - |  | - | - | - | - |  | - |
| 76 | 81 | N/A |  | - | - |  | - |  | - |  | - | - | - | - |  | - |
| 77 | 69 | 40 |  | - | - |  | - |  | - |  | - | - | - | - |  | - |
| 78 | 62 | 47 |  | - | - |  | - |  | - |  | - | - | - | - |  | - |
| 79 | 55 | 34 |  | - | - |  | - |  | - |  | - | - | - | - |  | - |
| 80 | 57 | 35 |  | - | - |  | - |  | - |  | - | - | - | - |  | - |
| 83 | 59 | 44 |  | - | - |  | - |  | - |  | - | - | - | - |  | - |

|  | Host |  |  | Symbiont |  |  | Polychaeta |  | Fish |  | Decapoda |  |  |  |  | Nudibranch |
| --- | --- | --- | --- | --- | --- | --- | --- | --- | --- | --- | --- | --- | --- | --- | --- | --- |
| Specimen | Tube length | Body volume |  | abudance | N of species |  | *Ophthalmonoe pettiboneae* |  | *Onuxodon fowleri* |  | *Eulenaios cometes* | *Polyonyx* cf. *heox* | *Polyonyx* sp. | *Tetrias* sp. |  | Tergipedidae gen.sp. |
| *Chaetopterus sp.* | |  |  |  |  |  |  |  |  |  |  |  |  |  |  |  |
| 84 | 64 | N/A |  | - | - |  | - |  | - |  | - | - | - | - |  | - |
| 85 | 59 | 51 |  | - | - |  | - |  | - |  | - | - | - | - |  | - |
| 86 | 76 | 45 |  | - | - |  | - |  | - |  | - | - | - | - |  | - |
| 87 | 41 | 23 |  | - | - |  | - |  | - |  | - | - | - | - |  | - |
| 89 | 78 | 43 |  | - | - |  | - |  | - |  | - | - | - | - |  | - |
| 90 | 56 | 39 |  | - | - |  | - |  | - |  | - | - | - | - |  | - |
| 91 | 52 | 33 |  | - | - |  | - |  | - |  | - | - | - | - |  | - |
| 92 | 56 | 36 |  | - | - |  | - |  | - |  | - | - | - | - |  | - |
