# Supplementary material for: Do syntopic host species harbour similar symbiotic communities? The case of Chaetopterus spp. (Annelida: Chaetopteridae)
Source: PeerJ. 2017 Feb 2;5:e2930. doi: 10.7717/peerj.2930 (PMC5292031; doi:10.7717/peerj.2930)
Supplement: Table S2 — Body volume (ml) and tube length (cm) of the 45 specimens of Chaetopterus cf. appendiculatus selected to estimate the relationship between these two measures in the analysis of community structure. [file peerj-05-2930-s002.docx]

| Specimen | Tube length | Body volume |  | Specimen | Tube length | Body volume |
| --- | --- | --- | --- | --- | --- | --- |
| 1 | 44 | 32 |  | 24 | 68 | 45 |
| 2 | 61 | 44 |  | 25 | 78 | 57 |
| 3 | 47 | 28 |  | 26 | 62 | 40 |
| 4 | 71 | 49 |  | 27 | 64 | 49 |
| 5 | 64 | 33 |  | 28 | 75 | 36 |
| 6 | 77 | 44 |  | 29 | 66 | 40 |
| 7 | 60 | 31 |  | 30 | 61 | 32 |
| 8 | 70 | 44 |  | 31 | 75 | 48 |
| 9 | 66 | 31 |  | 32 | 52 | 25 |
| 10 | 67 | 44 |  | 33 | 59 | 24 |
| 11 | 67 | 50 |  | 34 | 69 | 40 |
| 12 | 62 | 46 |  | 35 | 61 | 43 |
| 13 | 60 | 37 |  | 36 | 62 | 47 |
| 14 | 59 | 40 |  | 37 | 55 | 34 |
| 15 | 69 | 45 |  | 38 | 74 | 47 |
| 16 | 71 | 34 |  | 39 | 72 | 50 |
| 17 | 67 | 41 |  | 40 | 59 | 32 |
| 18 | 61 | 34 |  | 41 | 58 | 28 |
| 19 | 76 | 54 |  | 42 | 65 | 40 |
| 20 | 74 | 48 |  | 43 | 57 | 35 |
| 21 | 69 | 43 |  | 44 | 64 | 50 |
| 22 | 70 | 46 |  | 45 | 59 | 44 |
| 23 | 68 | 72 |  | 46 | 71 | 49 |
